# Crosstalk Between Type VI Secretion System and Mobile Genetic Elements

**DOI:** 10.3389/fmolb.2019.00126

**Published:** 2019-11-13

**Authors:** Arancha Peñil-Celis, M. Pilar Garcillán-Barcia

**Affiliations:** Instituto de Biomedicina y Biotecnología de Cantabria, Universidad de Cantabria—Consejo Superior de Investigaciones Científicas, Santander, Spain

**Keywords:** type VI secretion system, bacterial conjugation, horizontal gene transfer, cell-cell communication, T6SS regulation, mobile genetic elements, plasmids, integrative and conjugative elements

## Abstract

Many bacterial processes require cell-cell contacts. Such are the cases of bacterial conjugation, one of the main horizontal gene transfer mechanisms that physically spreads DNA, and the type VI secretion systems (T6SSs), which deploy antibacterial activity. Bacteria depend on conjugation to adapt to changing environments, while T6SS killing activity could pose a threat to mating partners. Here we review the experimental evidences of overlapping and interaction between the T6SSs, bacterial conjugation, and conjugative genetic elements.

## Introduction

Microbes often exist in complex multispecies communities where interaction is essential for keeping a balanced microbial ecosystem. Bacterial survival depends on the ability to succeed in the competition against rival bacterial cells. Critical to this goal is the type VI secretion system (T6SS), a macromolecular multiprotein complex dedicated to the delivery of toxins across the cell envelope of the donor bacteria (predator) into the cytoplasm of a target cell (prey), either another bacterium, or an eukaryotic cell [for recent reviews on T6SS structure, assembly, and activity, see (Cianfanelli et al., 2016; Nazarov et al., 2018; Nguyen et al., 2018; Cherrak et al., 2019; Wang et al., 2019). Only a few phyla of Gram-negative microorganisms encode T6SSs (Abby et al., 2016), suggesting a role for horizontal gene transfer (HGT) in its origin. Four T6SS phylogenetic subtypes are distinguished (Russell et al., 2014; Böck et al., 2017). Several protein complexes compose the T6SS most common subtype (T6SS^i^): a membrane complex (Durand et al., 2012, 2015; Rapisarda et al., 2019), which encompasses proteins TssJ, TssL, and TssM, a baseplate [TssE, TssF, TssG, and TssK (Nazarov et al., 2018; Park et al., 2018; Liebl et al., 2019)], a syringe (Wang et al., 2017), which is in turn composed of an inner tube that is secreted by the functional system [Hcp (Douzi et al., 2014)], a needle spike [VgrG (Uchida et al., 2014) and PAAR (Shneider et al., 2013)], and a sheath [TssB and TssC (Kudryashev et al., 2015)]. Furthermore, a sheath assembly protein, TssA (Schneider et al., 2019), and a sheath disassembly ATPase, TssH (also known as ClpV) (Pietrosiuk et al., 2011) are part of the system. A plethora of T6SS protein effectors and their corresponding immunity proteins have been identified (Durand et al., 2014; Kostiuk et al., 2017; Lien and Lai, 2017).

A claimed common evolutionary origin for T6SSs and contractile tailed-bacteriophages is supported by the conservation of some of their components (Hcp, VgrG, TssC, TssF, TssA, TssK, TssG, and TssE (Pukatzki et al., 2007; Leiman et al., 2009; Lossi et al., 2011; Planamente et al., 2016), and the similarities in the assembly mechanisms between the T6SS and the bacteriophage tail tube-sheath (Veesler and Cambillau, 2011; Lossi et al., 2013; Brunet et al., 2014). Accretion of DotU/IcmH and IcmF homologs of the Dot/Icm T4SS of *Legionella pneumophila* (Christie, 2016), TssL and TssM, which mediate the polar targeting of such T4SS (Ghosal et al., 2019), provides a docking station for the phage-like T66S device (Durand et al., 2015).

Most T6SSs are functional in a cell-cell contact-dependent way. Cell-cell communication mechanisms are thus expected to have an impact on T6SS activity. Several examples support the T6SS regulation by quorum sensing (QS) networks (Pena et al., 2019). A phosphorylation cascade that mediates a positive regulatory loop entwining T6SS and QS was found in *Vibrio alginolyticus* (Yang et al., 2018). In *Vibrio cholerae*, expression of Hcp showed to be growth phase-dependent, positively and negatively regulated by the QS regulators HapR and LuxO, respectively (Ishikawa et al., 2009; Zheng et al., 2010). Biofilm formation and T6SS expression in *Burkolderia cenocepacia* were upregulated by QS (Aubert et al., 2013), while expression of T6SS effector and immunity genes of *Burkholderia thailandensis* was also activated by QS (Majerczyk et al., 2016). In the case of *Pseudomonas aeruginosa*, its *three* T6SSs were differentially regulated by QS: whereas QS regulators LasR and MvfR suppressed the expression of H1-T6SS, they positively regulated that of H2- and H3-T6SS (Lesic et al., 2009). On the other hand, although cell-cell contact is not a requirement for natural transformation, in *Vibrio cholerae*, T6SS is part of the competence regulon and functions as a transformation enhancer for DNA acquisition (Borgeaud et al., 2015; Veening and Blokesch, 2017; Matthey et al., 2019). Competence-mediated induction of T6SS released DNA and made it accessible for HGT in *Vibrio cholerae*. Besides, competence-induced T6SS-mediated killing increased the natural transformation efficiency and boosted the acquisition of large genomic regions from killed neighbors (Borgeaud et al., 2015), including novel functional T6SS effector-immunity pairs (Thomas et al., 2017).

The above results suggest that T6SS and cell-cell communication processes are interwoven. Here we will focus on the contribution of another intercellular mechanism, bacterial conjugation, and its associated players, plasmids and integrative and conjugative elements (ICEs), to the activity of T6SS.

## T6SS Control by Plasmid-Encoded Regulators

**Figure 1:**
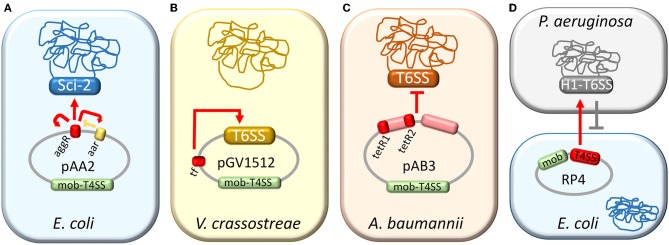
Control of T6SS activity by plasmids. Plasmid-mediated regulation of T6SSs in *E. coli* **(A)**, *V. crassostreae* **(B)**, *A. baumannii* **(C)**, and *P. aeruginosa* **(D)**. Plasmid regions involved in T6SS regulation are depicted in red. **(A)** pAA2-encoded auto-regulator AggR activates the Sci-2 T6SS (red arrows) and is negatively regulated by Aar (yellow lines). **(B)** Transcriptional factor (TF) of pGV1512 activates the plasmid-encoded T6SS (red arrow). **(C)** Two regions of the pAB3 plasmid, one of them including *tetR*-like regulators, repress the T6SS of *A. baumannii* (red lines) **(D)** Cell-cell interactions mediated by the T4SS of plasmid RP4 trigger H1-T6SS counterattack (red arrow), resulting in a decreased survival specifically in plasmid-bearing cells (gray lines).

T6SSs are implicated in a wide range of functions and regulated by a large diversity of mechanisms (Bernard et al., 2010; Alteri and Mobley, 2016). Transcriptional regulators of T6SS loci have been detected in plasmids. The first report on a T6SS regulation by a plasmid-encoded regulator was that of Sci-2 (Figure 1A). Sci-2 T6SS is encoded in entero-aggregative, avian-pathogenic and Shiga toxin-producing *Escherichia coli* strains (Journet and Cascales, 2016). It confers a growth advantage to entero-aggregative *E. coli* (EAEC) by causing non-immune *E. coli* killing (Brunet et al., 2013). The prototype EAEC strain 042 carries the pAA2 plasmid, which encodes a transcriptional regulator of the AraC family, the *aggR* gene (Nataro et al., 1994). AggR plays a central role in modulating adherence of EAEC 042 by activating plasmid-borne genes, such as the attachment adherence fimbriae *aafDA* (Elias et al., 1999), the anti-aggregative protein dispersin *aap* (Sheikh et al., 2002), and the T1SS *aatPABCD* for Aap transport (Nishi et al., 2003). EAEC chromosomal genes located in the pathogenicity island PAI-1 are also included in the AggR regulon (Dudley et al., 2006; Morin et al., 2013; Yasir et al., 2019). Among these genes, a cluster designated *aaiA-P* (Sci-2) was found expressed at least 2-fold higher in the wild-type strain 042 than in the *aggR-* derivative using a microarray approach (Dudley et al., 2006). Two proteins of this cluster were identified by mass spectrometry analysis in the whole-cell proteome of the wild-type strain, while they were absent in the *aggR*- derivative. One of these proteins, AaiC (Hcp) was also detected in the supernatant of exponentially-growing cells cultured in DMEM (Dudley et al., 2006), a condition that induces AggR production (Sheikh et al., 2001). An AggR-dependent promoter was localized in the region comprised from 466 bp upstream to 300 bp downstream of the predicted *aaiA* translational start site (Dudley et al., 2006). In the wild-type strain, the expression from this promoter was approximately 3-fold over that seen in the *aggR* mutant.

AggR was found to autoactivate its expression (Morin et al., 2010). By primer extension analysis, the transcriptional start site of the *aggR* promoter (P*aggR*) was located 40 nucleotides upstream of the translational start (Morin et al., 2010). DNA footprinting experiments revealed the presence of two AggR-binding sites: one upstream of the transcriptional start site and one downstream. A consensus sequence resembling sites for the Rns regulator from enterotoxigenic *E. coli* (Munson, 2013) was found for AggR binding: ANNNNNNTATC (Morin et al., 2010). EAEC plasmid and chromosomal AggR-regulated genes identified by RNAseq were found to contain a WWWWWWWTATC (where W means A or T) sequence spaced 21–23 bp upstream of the −10 promoter elements (Yasir et al., 2019). AggR is negatively controlled by the plasmid-encoded Aar repressor protein through direct binding (Santiago et al., 2016), while the *aar* gene is in turn positively regulated by AggR (Santiago et al., 2014). It remains unknown what environmental signal is sensed by AggR (Morin et al., 2013).

Another example of T6SS regulation by an AraC-like activator is provided by pGV1512, a conjugative plasmid that invaded *Vibrio crassostreae*, turning it from a benign oyster commensal into a pathogen (Bruto et al., 2017; Figure 1B). This plasmid encodes a T6SS organized into two divergently-transcribed operons. A plasmid region located between T6SS and T4SS, Px3, was found to be necessary for *V. crassostreae* virulence (Bruto et al., 2017). Px3 encodes a transcriptional regulator (TF) of the AraC family, which activated the transcription of plasmid-encoded T6SS genes, as shown by RNAseq analysis, and restored the virulence to a ΔPx3 mutant (Piel et al., 2019). Two functional promoters were located in the intergenic region between T6SS operons. Deletion of a palindromic sequence of six nucleotides spaced by five nucleotides, located between both promoters, altered the induction capacity of TF (Piel et al., 2019).

Negative transcriptional regulation exerted by a plasmid to a chromosomally-encoded T6SS has been also documented in *Acinetobacter baumannii* (Figure 1C). T6SSs are conserved and syntenic among *A. baumannii* strains (Weber et al., 2013). *A. baumannii* ATCC 17978 constitutively produces and secretes Hcp, a hallmark of an active T6SS, but initial efforts to observe a predator phenotype failed. There were no significant differences in the survival of any of the preys (*E. coli, A. baumannii, A. nosocomialis, A. baylyi*) in different experimental conditions tested (Weber et al., 2013). Nevertheless, when individual colonies from a clinical isolate of *A. baumannii* (strain Ab 04) were analyzed for Hcp secretion by ELISA, two contrasting profiles were found (Weber et al., 2015). Some colonies behaved coherently with a T6SS- and others with a T6SS+ phenotype. Whole-genome sequencing of both Ab 04 subpopulations revealed that their genomes encoded a T6SS, though they differed in the carriage of a multidrug-resistance conjugative plasmid (pAB04-1), absent in the cells exhibiting the T6SS+ phenotype.

This plasmid was not fully stable in *A. baumannii*, so a subpopulation lost it, rendering colonies that produced and secreted Hcp while becoming susceptible to antimicrobials. Colonies displaying the ±T6SS phenotypes were also isolated for *A. baumannii* strains 17978 and 1438 (Weber et al., 2015). In all cases, T6SS+ cells efficiently killed *E. coli* in competition assays, in contrast to their T6SS- counterparts. This killing was dependent on a functional T6SS, as verified by using mutants of essential T6SS components, which did not kill *E. coli*. Mating experiments in which the transmissible plasmid was transferred from donor *A. baumannii* cells with the T6SS- phenotype to T6SS+ recipient cells rendered transconjugants deficient in bacterial killing. These experiments provided evidence that pAB04-1 encoded the genetic determinants for silencing the *A. baumannii* T6SS activity.

The pAB04-1 backbone is commonly found in other *A. baumannii* strains, and the plasmid variants mainly differ in their antimicrobial-resistance cargoes (Weber et al., 2015). *A. baumannii* prototype strain, ATCC 17978, harbors a pAB04-1-like plasmid, pAB3. Transferred to either a clonally-unrelated *A. baumannii* strain, *A. baylyi* or *A. nosocomialis*, the transconjugants did not secrete Hcp, indicating that the T6SSs of these strains were also susceptible to repression by the plasmid and thus pointing to a common repression mechanism in a broad range of *Acinetobacter* species (Di Venanzio et al., 2019b). Another pAB04-1-like plasmid, pAB5, from *A. baumannii* strain UPAB1, was shown to regulate the expression of multiple chromosomally-encoded virulence factors, including T6SS (Di Venanzio et al., 2019a). An UPAB1 derivative lacking pAB5 displayed increased susceptibility to multiple antibiotics and activation of the T6SS, as observed by secretion of the T6SS-associated protein Hcp. Two *tetR*-like regulators were found to be encoded only in pAB04-1-like plasmids (Weber et al., 2015). The deletion of either pAB3 *tetR* gene or both increased Hcp expression but did not restore Hcp secretion (Di Venanzio et al., 2019b). The deletion of the *tetR*-like repressor genes was thus not enough to trigger *per se* the T6SS activity. Nevertheless, when these regulators were overexpressed in a T6SS+ phenotype ATCC 17978 population, Hcp expression and secretion drastically decreased, and this transformed population was impaired as a predator in its *E. coli* killing ability (Weber et al., 2015).

## T6SS Activity Affects Bacterial Conjugation

*P. aeruginosa* senses exogenous attacks by the T6SS of akin and non-akin bacteria and post-translationally activates its H1-T6SS at the precise location of the initial strikes (Basler and Mekalanos, 2012; Basler et al., 2013). Other perturbations of the cell envelope, such as the presence of the Gram-negative bacterial membrane disruptor polymyxin B, also increased the H1-T6SS activity (Ho et al., 2013). These results suggested that membrane perturbations trigger the H1-T6SS activity.

Bacterial conjugation is an HGT mechanism that involves contact between the cell envelopes of the mating-pair partners, i.e., donor and recipient cells. It is essentially mediated by plasmids and integrative and conjugative elements (ICEs) (Smillie et al., 2010; Guglielmini et al., 2011). These genomic platforms encode the genetic requirements for their transfer: a *mob* region containing an origin of transfer (the single element strictly required *in cis*), a relaxase (Smillie et al., 2010; Zechner et al., 2017; Guzmán-Herrador and Llosa, 2019), and a type IV coupling protein (Gomis-Rüth et al., 2004; Peña and Arechaga, 2013); and a mating-pair bridge composed of a type IV secretion system (T4SS) (Cabezón et al., 2015; Bergé et al., 2017; Grohmann et al., 2018; Li et al., 2019).

Donor *E. coli* strains bearing broad host-range conjugative plasmids RP4/RK2 or pKM101, but not the narrow host-range plasmid F, were more sensitive to killing by a T6SS+ *P. aeruginosa* recipient than plasmid-lacking donors (Ho et al., 2013; Figure 1D). Furthermore, RP4-containing *E. coli* donors were selectively killed by *P. aeruginosa* in a mixed culture with RP4-lacking *E. coli* cells. *P. aeruginosa* mutants defective in the attack-sensing pathway genes *tagT* and *pppA* exhibited greater conjugation efficiency as recipient strains and did not kill RP4-bearing *E. coli* donors. Transposon mutagenesis of RP4 rendered T4SS mutants with impaired ability to transfer and induce T6SS donor-directed killing response in *P. aeruginosa*. RP4 mutants in the relaxosome components genes *traI* and *traJ* and the coupling protein gene *traG* were also defective in DNA conjugation but showed increased donor-directed T6SS response compared to wild type RP4, suggesting that successful DNA transfer was not necessary to activate a T6SS attack by *P. aeruginosa*. So, mating donors can trigger a T6SS response in the recipient, which in turn causes a decrease in their survival.

In the case of *A. baumannii*, conjugative dissemination of the pAB4-01/pAB3 plasmids relies on the repression of the T6SS encoded in the donor strain. To evaluate the impact of the plasmid-mediated T6SS repression in plasmid dissemination through conjugation, pAB3 derivatives were used in mating assays (Di Venanzio et al., 2019b). One of them, pAB3Δt*etR1,2* lacked genes ACX60_RS18875-ACX60_RS18900 (GenBank Acc. No. NZ_CP012005.1), a region that contains the *tetR*-like genes *tetR1* and *tetR2*. On this mutant, a second deletion comprising genes ACX60_RS18760-ACX60_RS18795 was introduced, producing plasmid pAB3^*^. Wild-type and mutant plasmids were efficiently transferred between isogenic *A. baumannii* ATCC 17,978 strains (T6SS-resistant). When a non-immunogenic and thus T6SS-susceptible ATCC 17978 derivative was used as a recipient, pAB3 and pAB3Δt*etR1,2* transfer efficiencies were not altered. Nevertheless, the transfer of the mutant plasmid pAB3^*^ and that of mobilizable plasmids relying on pAB3^*^ as a helper were practically abolished due to recipient killing mediated by the donor's T6SS. Congruently, in competition experiments pAB3 overcame the mutant plasmid pAB3^*^ at invading a T6SS-susceptible population. On the other hand, the efficiency of plasmid conjugation was also deeply affected when the recipient strain was T6SS-proficient and non-isogenic to the donor. pAB3-like plasmids would thus seem to guarantee their transmission by preventing T6SS-mediated killing of non-isogenic recipient strains, depending on their capacity to repress T6SS activity in the donor cells.

## T6SSs Encoded in Mobile Platforms

The above results point to T6SS and conjugation as incompatible processes. Plasmids and ICEs are mobile genetic platforms that rely on bacterial conjugation for their dissemination, and thus do not seem *a priori* good platforms for T6SS. Plasmids have a predominant role as genetic couriers (Halary et al., 2010). Twenty-nine plasmids encoding T6SS genes are listed in the T6SS database SecReT6 (http://db-mml.sjtu.edu.cn/SecReT6/) (Li et al., 2015). Experimental evidence on these T6SSs is available only for that encoded in the 2.1 Mb megaplasmid pGMI1000MP of the plant pathogen *Ralstonia solanacearum* GMI1000. A strain containing a *tssB* mutant of this plasmid was reported to be impaired in Hcp secretion and biofilm formation (Zhang et al., 2014). It had also significantly attenuated its virulence on tomato plants. Megaplasmids from other *R. solanacearum* strains also encode T6SS genes: RCFBPv3_mp, CMR15_mp, FQY_4 megaplasmid, Po82 megaplasmid, and mpPSI07. Plasmids pESA3 (*Cronobacter sakazakii*) and pEA320 (*Pantoea ananatis*) are also recorded in SecReT6. The existence of plasmids encoding T6SS genes in these species does not seem anecdotal. Using PCR probes based on the T6SS of pESA3, at least a partial T6SS cluster was detected in 175 out of 177 plasmid-harboring *C. sakazakii* strains analyzed (Franco et al., 2011) and comparative genomics of T6SSs in strains of *P. ananatis* from different environments revealed a plasmid-borne T6SS in a third of the analyzed strains (Shyntum et al., 2014). This T6SS type was restricted to strains of *P. ananatis* isolated from symptomatic plant material, suggesting the possibility of an association between the plasmid-borne T6SS and either pathogenicity or host specificity.

Out of the SecReT6 database, and besides the above-mentioned in pGV1512, a functional T6SS was reported in the *Rizobium etli* Mim1 megaplasmid pRETMiM1f (Salinero-Lanzarote et al., 2019). Immunodetection of Hcp protein indicated that this T6SS was active at high cell densities, in the presence of root exudates, and in bean nodules. *R. etli* T6SS- mutants produced plants with lower dry weight and smaller nodules than the wild-type strain, indicating for the first time that the T6SS played a positive role in *Rhizobium*-legume symbiosis.

ICEs are the most abundant conjugative elements in practically all prokaryotic clades (Guglielmini et al., 2011). Genome analysis strongly suggested transfer of the T6SS^iii^ subtype mediated by an ICE between Bacteroidales strains within the human gut ecosystem (Coyne et al., 2014). T6SS^iii^ has been exclusively found in Bacteroidetes (Abby et al., 2016) and functions in a mechanistically similar manner to T6SS^i^ to target competitor bacteria (Russell et al., 2014). T6SS^iii^ loci were found in more than half of human gut Bacteroidales strains, and they segregated into three evolutionarily-distinct genetic architectures, two of which were located on ICEs, and one of them had been transferred among co-resident Bacteroidales species in the human gut (Coyne et al., 2016). Low diversity in the effector-immunity pairs of the ICE-encoded T6SS^iii^ was detected in human microbiome samples, an indicator of neighbor compatibility likely facilitated by HGT through ICE conjugation (Verster et al., 2017).

## Conclusions

The antibacterial activity of T6SS affects bacterial conjugation by either killing plasmid-bearing donors or non-immune recipients and thus could undermine the DNA spreading. In turn, conjugative platforms (plasmids and ICEs) encode regulatory elements for controlling the T6SS activity. More intriguing is the fact that T6SS is not confined to the chromosome, but also present in these mobile platforms. How plasmids and ICEs have found their ways to deal with this weaponry carriage without sacrificing their transfer potential remains to be researched.

## Author Contributions

MG-B conceived the study. AP-C and MG-B wrote the manuscript.

### Conflict of Interest

The authors declare that the research was conducted in the absence of any commercial or financial relationships that could be construed as a potential conflict of interest.
